# Five A’s counseling in weight management of obese patients in primary care: a cluster-randomized controlled trial (INTERACT)

**DOI:** 10.1186/s12875-018-0785-7

**Published:** 2018-06-23

**Authors:** Franziska D. Welzel, Janine Stein, Alexander Pabst, Melanie Luppa, Anette Kersting, Matthias Blüher, Claudia Luck-Sikorski, Hans-Helmut König, Steffi G. Riedel-Heller

**Affiliations:** 1 Institute of Social Medicine, Occupational Health and Public Health, Medical Faculty, University of Leipzig, Philipp-Rosenthal-Straße 55, 04103 Leipzig, Germany; 2 Integrated Research and Treatment Centre (IFB) AdiposityDiseases, University Hospital Leipzig, Leipzig, Germany; 3 Clinic for Psychosomatic Medicine and Psychotherapy, University Hospital Leipzig, University of Leipzig, Leipzig, Germany; 4 University of Applied Sciences SRH Gera, Gera, Germany; 5 Department of Health Economics and Health Services Research, University Medical Centre Hamburg-Eppendorf, Hamburg, Germany; 6 Institute of General Medicine, University of Leipzig, Leipzig, Germany

**Keywords:** Obesity, 5As counseling, Primary care, Doctor-patient-interaction, cRCT

## Abstract

**Background:**

Obesity is one of the most prevalent health problems in western societies. However, it seems not effectively managed in the healthcare system at present. Originating from smoking cessation a tool called the 5As for obesity management has been drafted and adapted by the Canadian Obesity Network (CON) to improve weight counseling and provider-patient-interaction. This paper describes the rationale and design of the INTERACT study. The objective of the INTERACT study is to evaluate the effectiveness and intervention costs of a 5As eLearning program for obesity management aimed specifically at general practitioners (GPs).

**Methods:**

The INTERACT study is a cluster randomized controlled trial aimed at implementing and evaluating an online-tutorial for obesity management based on the 5As approach in cooperating primary health care practices. Effectiveness of the 5As intervention will be evaluated by assessing patients and doctors perspectives on obesity management in primary care before and after the training. GPs in the intervention group will get access to the 5As obesity management online-tutorial while GPs in the control group will be assigned to a waiting list. Outcome measures for patients and GPs will be compared between the intervention group (treatment as usual + training of the GP) and the control group (treatment as usual). Hierarchical regression models will be used to analyze effects over time pre- and post-intervention.

**Discussion:**

The 5As present physicians with a simple mnemonic for patient counseling in the primary care context. While the use of the 5As in weight counseling seems to be associated with improved doctor-patient interaction and motivation to lose weight, intervention studies assessing the effectiveness of a short 5A eLearning tutorial for physicians on secondary outcomes, such as weight development, are lacking.

**Trial registration:**

The study has been registered at the German Clinical Trials Register (DRKS00009241; date of registration: 03.02.2016).

## Background

Obesity, defined by a body mass index (BMI) greater than 30 kg/m^2^, is highly prevalent in western countries. According to the World Health Organization (WHO), the worldwide prevalence of obesity has more than doubled within the last 30 years [1]. In Germany, a national survey revealed that 23% of all men and 24% of all women are obese [2]. The consequences of obesity are manifold ranging from severe somatic diseases (e.g. type 2 diabetes, obstructive sleep apnea, cardiovascular disease) and psychiatric co-morbidities [3] to psychosocial impairment in form of stigmatization and discrimination [4–6]. Obesity prevention and treatment have therefore been put on the agendas of the WHO [7].

Primary care physicians have a crucial role when designing weight management programs as they see their patients on a regular basis and over a long period of time [8]. Additionally, studies showed that patients who wish to reduce weight based on their doctor’s advice reduce more weight and can be motivated more by their general practitioner (GP) [9, 10]. However, patients themselves are not likely to address obesity and weight reduction with their GP and may not even consider their GP as ideal person to support them in weight management goals [11]. In fact, patients may even perceive their attending GPs to be responsible for initiating the discussion about weight and weight management [12]. Studies in the German health care system indicate that weight management in primary care is unsatisfactory in several aspects. Aside from being under-recognized [8], obesity in primary care patients is often not addressed by the physicians [13]. Other research showed that conservational strategies are seen most effective and therefore recommended far more often by health care professionals [14, 15]. However, even these strategies seem not consistently discussed with patients. Barriers include negative attitudes towards patients with obesity, a lack of knowledge of the overall pathophysiology of obesity and familiarity with current guidelines on the management of obesity, as well as a lack of self-efficacy in weight counseling [15–17].

Originated from smoking cessation counseling, a tool called the 5As (assess risk, ask about readiness to lose weight, advise change, assist in establishing interventions and securing goal attainment and arrange follow up) has been proposed to be adapted and used in weight counseling [18]. In smoking cessation, the use of the 5As has been successfully promoted and tested [19, 20]. A positive impact on quit rates and motivation has been reported after the transfer to the primary care setting [19].

Studies using the 5As in weight loss settings show that their use is positively correlated with patient motivation and intention to lose weight [21]. While some GPs already intuitively rely on components of the 5As in weight counseling, studies analyzing the quality of physician-patient encounters show a lack of consistency and a failure to include all 5As in counseling [22, 23]. The Canadian Obesity Network (CON) has set out to develop a standardized framework for the use of the 5As in weight counseling that combines the Canadian Obesity Guidelines [24] with the structural framework of the 5As and consists of hand-out material and an online tutorial [25]. All components (ask, assess, advise, agree, assist) have been elaborated in detail and provide clear guidance in counseling [26]. A pilot study showed a positive impact on quality of counseling and provider-patient-communication in the short term [27]. Results from long-term studies, however, are lacking.

### Objectives

The objective of the INTERACT study is to evaluate the effectiveness and intervention costs of an online-tutorial implementation for obesity management in primary care. The online-tutorial aims at increasing the quantity and quality of provider-patient interaction regarding weight management in patients with obesity and improving patient motivation and weight management goals. The aim of this study is to evaluate the online-intervention on the quality of the provider-patient-interaction.

## Methods/design

### Study design and study setting

The INTERACT-study comprises a cluster randomized controlled trial (cRCT) to implement and evaluate the effectiveness of an e-learning program (the 5As tutorial) for obesity management aimed specifically at GPs. The trial will be conducted within the primary care setting in the region of central Germany. The Institute of Social Medicine, Occupational Health and Public Health of the University of Leipzig (ISAP) has established a primary care physician network that consists of collaborating GPs. Based on this network, primary care physicians will be contacted and invited to participate in the study. The Interact trial includes patients with obesity recruited from cooperating GPs. Primary efficacy endpoint will be the assessment of the provider-patient interaction regarding obesity and weight management from the patients’ perspective.

### Intervention scheme

**Fig. 1:**
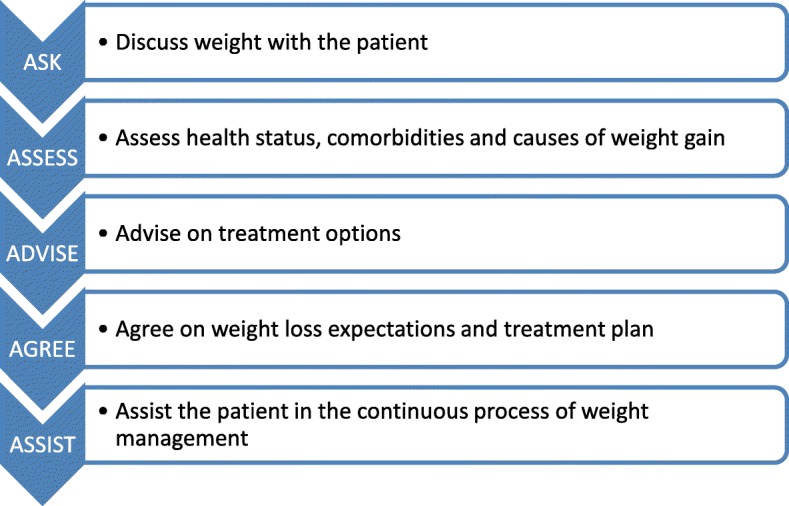
Overview of the 5As for obesity management [25]

**Fig. 2:**
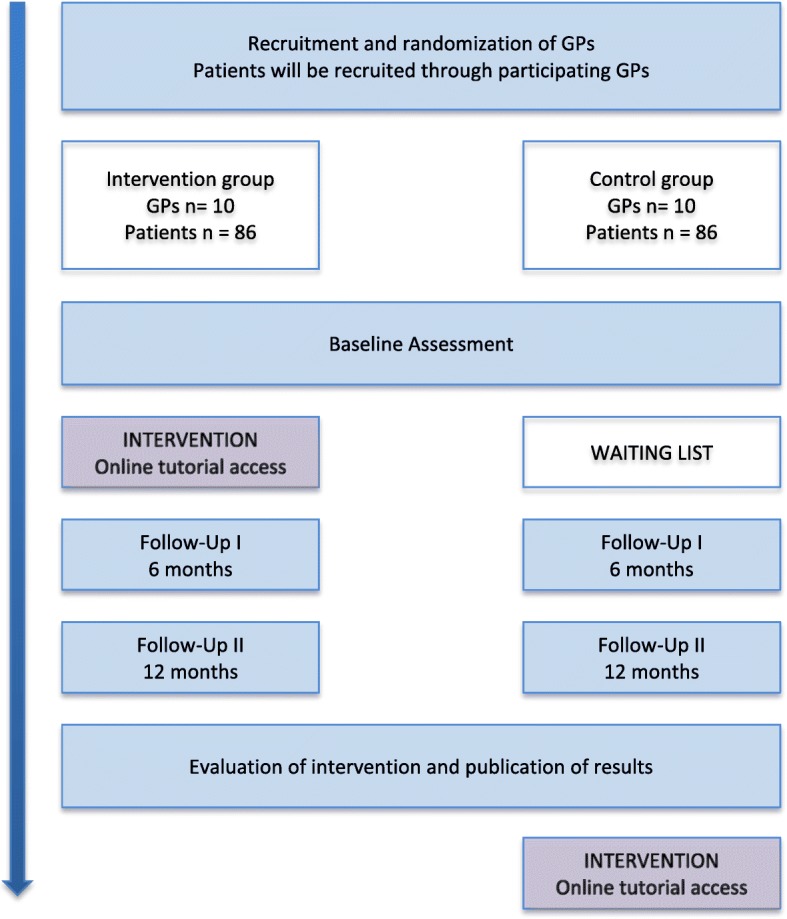
Cluster-randomized trial intervention scheme

For the present study, we have developed a 30 min online-tutorial based on the 5As framework of obesity management by the CON which covers recommendations on how to discuss weight with the patient (ASK), assess obesity related risks and causes of weight gain (ASSESS), advise on treatment options (ADVISE), agree on weight loss expectations and treatment plan (AGREE) and assist the patient in the ongoing process of losing weight (ASISST). The 5As framework has the potential to improve weight management counselling to patients by encouraging GPs to start sensitive conversations with the patient and achieve agreement on weight management goals and strategies. A key principle of the 5As framework is to measure the success of weight management in improvements in overall health and well-being rather than in the amount of weight loss. The 5As online-tutorial includes five knowledge sections that cover the 5As and the current state of knowledge on obesity management. The online-tutorial contains a short knowledge quiz at the end. An overview of the 5As is provided in Fig. 1. The 5As online-tutorial will be implemented in cooperating primary health care practices. Participating GP practices will be randomly allocated either to the intervention group with continuous access to the 5As online tutorial or to the control group, where GPs will be assigned to a waiting list condition. Waiting list practices will gain access to the 5As online-tutorial 6 months after the trial ended. GPs of the intervention group will be sent access data to the 5As online-tutorial after return of the baseline questionnaire (BL) and will have continuous access to the 5As program throughout the trial. Intervention completion by GPs of the intervention group will be checked and GPs will be reminded to complete the 5As intervention within two months after gaining access to the tutorial. CME points (Continuing Medical Education) will be granted after completion of the intervention module. Patients with obesity will be recruited in cooperation with participating GP practices. Patients of both groups (intervention group, control group) will be assessed using comprehensive questionnaires at recruitment (baseline, BL) as well as 6 and 12 months follow-up (after recruitment). GPs of both groups will be asked to complete questionnaires at BL (after recruitment) and 12 months follow-up. Furthermore, GPs of both groups will be asked to assess the health and weight status of each of their recruited patients immediately after patient inclusion and 12 months after patient inclusion. Participating GPs and patients will be invited to complete the follow-up assessments by postal reminder. A flow diagram of the overall intervention scheme is provided in Fig. 2.

### Inclusion/ exclusion criteria

No exclusion or inclusion criteria apply for the GPs. Patients will be recruited if they fall within the BMI and age range (BMI greater than 30 kg/m^2^, age 18 to 60 years) but do not present at the GPs with an acute illness that needs priority management. The criteria for inclusion and exclusion of participants are summarized in Table 1.

**Table 1:** Criteria for inclusion and exclusion of participants

Criteria for inclusion:	
- Body mass index greater than 30 kg/m^2^	
- Age between 18 and 60 years	
- German as native language	
Criteria for exclusion:	
- Acute physical or mental illness that needs priority management and make study participation impossible according to the GP	

### Randomization and blinding

GPs, who agree to participate, will be sequentially allocated to either the intervention or the control group by a trained staff member at the Institute of Social Medicine, Occupational Health and Public Health (ISAP) external to the project. Randomization will be done by adaptive randomization [28] applying the biased coin method and using a computerized random number generator. Given that the intervention is addressed at the practitioners, it is not possible to blind GPs to their group allocation. However, GPs will be blinded to knowledge of the primary outcome measures. Patients will be blinded with regard to the group allocation. Participating GPs will be informed about allocation to control or intervention group after randomization completion by postal mail including a sealed envelope with the assigned intervention category. They will further be sent recruitment material and consent forms for patients by postal mail. In addition, GPs will receive explanatory instructions about the recruitment material and the recruitment process by telephone through a research assistant who will not be involved with tracking of patient enrollment or data collection. GPs will receive an instruction how to recruit patients in a blinded manner. Practitioners will identify and recruit patients according to the inclusion and exclusion criteria within their practices. They will inform eligible patients about the study and ask them to participate. Information material and consent forms for patients are identical for intervention and control group. The statistician analyzing the data will be unaware of the group allocation. The investigator and the research team will remain blinded to the results until data collection is completed.

### Outcomes and measures

#### General practitioners

GPs will be asked to complete two short pre- and post-intervention questionnaires. The pre-intervention questionnaire will be filled out upon recruitment (BL). The post-intervention questionnaire will be sent by postal mail 12 months after BL. Pre- and post-intervention questionnaires will contain closed questions on referral and counseling behavior of patients with obesity, satisfaction with own knowledge about obesity, attitudes towards obesity as a chronic disease, relevance of different causes of weight gain and attitudes on different aspects of obesity management. Stigma concerning obesity will be assessed using the Fat Phobia Scale (FPS) by Bacon et al. [29]. The FPS consists of 16 pairs of adjectives on a semantic differential. Furthermore, GPs of the intervention group will be asked to evaluate the 5As online tutorial in regards to the relevance of its knowledge contents and its usability within the primary care setting 12 months after intervention completion.

Furthermore GPs will be provided with a list of co-morbid conditions and will be asked to complete this list and assess the weight status for each participating patient at BL and 12 months follow-up.

#### Patients

Patients will be asked to complete three questionnaires at recruitment as well as 6 months and 12 months follow-up, respectively. All three questionnaires will be sent by postal mail. In addition to demographics (gender, age, educational level, working status, marital status), the outcome measures for patients will include patient-doctor interaction regarding obesity management over the past 6 months, quality of life, weight history and BMI, motivation to engage in weight management strategies, self-stigma regarding obesity, the assessment of depressive and anxiety symptoms and personality traits. Instruments for each of the mentioned outcomes are described in the subsections below. As the 5As framework primarily aims to improve weight management counselling by improving the interaction between patients with obesity and their physicians the primary outcome of this study will focus on the patient-doctor interaction. Changes in weight will be assessed as a second outcome after investigating improvements in the patient-doctor interaction regarding weight counselling.

##### Primary outcomes

Patient’s perspective on the *patient-doctor interaction* regarding the management of obesity over the past 6 months will be assessed using an adapted version of the German adaptation of the Patient Assessment of Chronic Illness Care (PACIC) [30, 31]. The PACIC is a 26-item questionnaire scored on a 5-point Likert scale ranging from 1 (= never) to 5 (= always). The first 20 items of the PACIC are arranged into five scales: patient activation, delivery of care, goal setting, problem solving, and follow-up. Further six items of the PACIC cover elements of the original 5As approach (ask, advise, agree, assist, arrange). The 5A PACIC sum score will be calculated according to the scoring instruction by Rosemann et al. [31]. Higher scores indicate a stronger congruency to the 5A approach.

##### Secondary outcomes

*Quality of life* will be assessed using the EQ-5D-5 L [32] which consists of a visual analogue scale to assess health related quality of life on a scale from 0 (= worst health) to 100% (= best health). Additionally, five questions cover health related quality of life and perceived impairments rated on a five-point scale from 0 to 4.

*Weight history* will be assessed at BL including the assessment of the beginning of obesity, the number of previous weight loss attempts and the types of weight loss attempts. The *BMI* will be calculated based on self-reported height and weight. Height will only be determined at the baseline assessment, whereas weight will be assessed at every follow-up. In addition, weight will be assessed through the attending GP at BL and 12 month follow-up. As in previous research, a stable weight is defined not differing more than 5% of the original body weight [33].

The *willingness to engage in weight management strategies* will be measured using the Readiness Ruler adapted from Zimmermann et al. [34] and Stott et al. [35]. The ruler is a visual analogue scale to assess the readiness for change regarding weight loss on a scale from 0 (= not ready to change) to 10 (= ready to change). Additionally, *weight loss intentions and current activities of weight management* will be assessed using the stages of change algorithm adapted from DiClemente and Prochaska [36] and Prochaska et al. [37]. The stages of change algorithm consists of four questions according to which individuals are categorized into four separate categories of change (precontemplation, contemplation, action and maintenance).

*Depressive symptomatology* within the last two weeks will be measured using the German adaptation of the PHQ-9 [38] which comprises 9 items with a four-point response scale from 1 (=not at all present) to 4 (=present almost every day). The sum score of the PHQ-9 allows classifying the severity of the depressive symptomatology into the following categories: no depression, mild depression, pronounced depression and severe depression.

*Self-stigma* will be measured using a German adaptation of the Weight Bias Internalization Scale (WBIS) [39, 40]. The WBIS comprises 11 items and was developed to assess internalized weight bias among individuals with obesity.

##### Other measures/ covariates

To control for potential confounding effects the following measures will be assessed:

*Anxiety symptoms* within the last four weeks will be measured using the GAD-7 [41] which comprises 7 items with a three-point response scale from 1 (=not at all present) to 3 (=present for more than half the days). The sum of the scale allows classifying the severity of anxiety symptoms into: marginal anxiety, mild anxiety, pronounced anxiety and severe anxiety. The *panic syndrome* will be assessed using the panic-syndrome module of the PHQ-D (phq3a-phq4k) [42]. The panic syndrome module comprises 15 items with a yes/no response scale.

*Personality traits* will be assessed using the 10-Item Big Five Inventory (BFI-10) [43] which is an abbreviated version of the BFI-44. The BFI-10 comprises two items for each of the five personality dimensions with a five-point response scale from 1 (=disagree strongly) to 5 (=agree strongly).

Additionally, four half-open questions cover the *counseling experience of patients* within the last six months with their attending GP (“Have you seen your GP within the last six months?”, “How often have you seen your GP within the last six months? Please consider only direct contacts with your GP”, “Has your weight been discussed in the consultation and who took the initiative?”, “Which aspects of weight have been discussed?”).

### Data collection and data management

Data will be collected using questionnaires sent by postal mail including a prepaid return envelope. Monetary incentives (10 Euros per questionnaire) are used to ensure high follow up rates. Double data entry will be used for all questionnaires to keep the rate of data errors very low. Since the trial is based on questionnaires, monitoring is not an issue. The nature of this trial renders a data monitoring and safety board unnecessary. Completeness of the study data and study documents will be audited by the trial manager.

### Data analysis

After completion of the trial data cleaning and quality control will be conducted. Prior to testing the effectiveness of the intervention, appropriate statistical tests will be used to examine possible baseline differences between groups (e.g. age, sex, educational level, employment status and marital status). Hierarchical regression models will be used to analyze treatment effects from baseline to follow up. Models will be adjusted for the baseline outcome value and confounding factors, such as age, sex and socio-economic background. All analyses will be intention-to-treat in order to control for non-random dropout effects. Patterns of missing values will be inspected and, if applicable, replaced using appropriate statistical methods. Additionally, the effect of the intervention on provider knowledge, satisfaction with own knowledge and perceived counseling behavior will be analyzed. The level of statistical significance will be set at *p* < 0.05 for all statistics.

#### Intervention costs

The intervention costs will be analyzed considering one-off development costs and running costs for the implementation of the 5A online tutorial. Development costs consisted of labor costs and administrative expenses (license and service fees, technical implementation). Monetary valuation of the labor costs will be based on the collective labor agreement for Federal States (TV-L). Administrative expenses will be documented within the framework of the study. Running costs consist of labor costs for the implementation of the 5A online tutorial by GPs and administrative expenses (technical support, system maintenance and backup). Monetary valuation of the labor costs for GPs will be based on the unit cost for GP contacts and the actual time expenditure of GPs.

#### Power calculation and sample size

Based on previous research, a power based sample size calculation was done using Stata 13.1 SE software package (StataCorp LP, College Station, TX). The primary outcome measure, the 5A PACIC sum score, may yield a minimum of 15 points difference prior to post-intervention. With a power of 95% and a standard deviation of 20 points [27], a sample size of *n* = 47 participating patients for each group was estimated to detect group differences. Given a 30% drop-out-rate, which is based on previous work of the ISAP, the overall sample size would comprise *n* = 134. According to Rueda-Clausen et al. [27] 15 points were regarded as a clinical significant change in the 5A PACIC sum score. Taking into account the interclass correlation coefficient of 5% from the cluster randomization the sample size would increase slightly to *n* = 66 patients per group. Given the 30% drop-out-rate *n* = 86 patients were estimated for each group. We estimate that 6–7 patients per practice could be recruited into the trial. Therefore, the trial aims to enroll at least 20 GPs. The overall sample will comprise 172 patients and not less than 20 GPs.

### Ethical considerations

The ethics committee of the University of Leipzig has approved this study. The study will be performed in accordance with the Guidelines for Good Clinical Practice (ICH-GCP), the Declaration of Helsinki in its latest version and international and local laws. Written informed consent from participating subjects will be obtained. Only subjects will be included who provided valid informed consent. Participants are provided a telephone contact in case of further enquiry. The intervention aiming at improved doctor-patient interaction is non-invasive and does not carry any specific risks to participants. Furthermore, measures will be established to ensure that ethical regulations are being fully complied with during the entire course of the study. All participants will be assigned an individual identification code. The data analyst and primary investigators will have access to the final data set. Data will be blinded of any identifying participant information. The appropriate regulations of local data legislation will be fulfilled in its entirety. The implementation of the 5As for obesity management was officially licensed by the CON. Protocol modifications will be communicated to all relevant parties.

## Discussion

Although a number of practical guidelines for the treatment of obesity exist, the quality of weight counseling in primary care seems to be unsatisfactory in several aspects [8, 13]. Obesity management in primary care is therefore worthy of improvement [15–17]. However, GPs are usually challenged by heavy workloads and the delivery of health care is bound to tight time constraints. Given differing demands, strategies to improve GPs counseling abilities need to be easy to use and time effective. The 5As are a simple mnemonic and considered a patient-centered counseling tool to encourage patients to change behavioral habits. Studies assessing the use and the effectiveness of the 5As in the context of weight counseling found that physicians primarily use some form of “Assess” and “Advise” or “Ask”, while they rarely apply the essential components of “Assist”, “Agree” and “Arrange” [21, 22]. Jay et al. [21] reported that the use of the 5As and especially the number of 5As used by physicians’ was associated with increased patient motivation to engage in weight management strategies. Further research has shown beneficial effects of the 5As in weight counseling on patients motivation to change exercise and eating patterns [22, 27]. However, most studies investigating the effects of the 5As in weight counseling had a cross-sectional design and did not measure direction of the effects observed or actual behavior change. Cross-sectional studies provide a snapshot of the physicians’ use of the 5As and its influence on patient motivation. Still, the chronic nature of obesity and overweight may need longitudinal research to investigate how the quality of weight counseling contributes to actual long-term health benefits in patients with obesity or overweight. There is a clear need for long-term studies investigating the effects of the 5As on doctor-patient interaction, patient motivation and weight development and compare those to a control group with standard treatment only. In the present study, the evaluation of changes in the quality of care for patients with obesity will be based on a validated instrument suitable for trials assessing the doctor-patient interaction. The PACIC 5 A is considered a comprehensive instrument with good reliability and validity [31] and offers a little time consuming measure to assess the quality of care for patients with chronic diseases. Furthermore, open-ended questions will be included in the assessment of GPs of the intervention group to allow for possible qualitative data analysis on GPs experience with the e-learning program.

### Strengths

As this study uses an cRCT-design it can add substantially to the knowledge of how the implementation of a short 5As educational training may contribute to improved weight management as perceived by patients and physicians. As method of randomization we decided to use the procedure of adaptive randomization by applying the biased coin method. Biased coin randomization provides a valuable feature in small to moderate sized studies to reduce probability of imbalance between intervention and control group and is considered to be acceptable with regard to unpredictability of group allocation [44]. This method is preferred over other randomization techniques (e.g. blocking) because it facilitates quick patient recruitment and may prevent withdrawal of already recruited GPs. Furthermore, the 5As intervention of this study will be delivered via eLearning offering a convenient way of educational training which can easily be incorporated in the busy workloads of GPs. The use of eLearning programs in delivering educational training in the health professions has gained interest within the last years and has been shown to be comparable to traditional methods (e.g. lecture-based approaches) in terms of effectiveness [45–47]. To our knowledge, this will be the first study to implement a 5As eLearning intervention for obesity counseling as part of a cRCT-study within the German primary health care setting.

### Conclusion and dissemination

The 5As propose a theory-driven, feasible tool for weight management in primary care that has proven its value in smoking cessation and addiction counseling. A transfer to weight management has been achieved and an implementation and evaluation in the German health care system is now needed. This intervention study therefore contributes to this goal and expands the present research on the key role of promoting weight management to the German primary care context. This research project provides materials that will be disseminated Germany-wide after its completion. Dissemination will be assured by approaching national key players, such as the German Obesity Association (DAG) and the Association of Primary Care Physicians (DEGAM). After successful evaluation, the 5As online tool will be made accessible to providers in relevant networks and support local management of patients with obesity.

## Abbreviations

BFI: Big Five Inventory
BL: Baseline
BMI: Body Mass Index
CON: Canadian Obesity Network
cRCT: cluster Randomized Controlled Trial
DAG: German Obesity Association
DEGAM: German Association of Primary Care Physicians
FPS: Fat Phobia Scale
GP: General Practitioner
ISAP: Institute of Social Medicine, Occupational Health and Public Health of the University of Leipzig
PACIC: Patient Assessment of Chronic Illness Care
WBIS: Weight Bias Internalization Scale
WHO: World Health Organization
